# Evaluation of the Structure and Health Impacts of Exercise-Based Cardiac and Pulmonary Rehabilitation and Prehabilitation for Individuals With Cancer: A Systematic Review and Meta-Analysis

**DOI:** 10.3389/fcvm.2021.739473

**Published:** 2021-09-22

**Authors:** Julia N. Rickard, Arun Eswaran, Stephanie D. Small, Alis Bonsignore, Maureen Pakosh, Paul Oh, Amy A. Kirkham

**Affiliations:** ^1^Faculty of Kinesiology & Physical Education, University of Toronto, Toronto, ON, Canada; ^2^Lawrence S Bloomberg Faculty of Nursing, University of Toronto, Toronto, ON, Canada; ^3^Library & Information Services, Toronto Rehabilitation Institute, Toronto, ON, Canada; ^4^Cardiovascular Prevention and Rehabilitation Program, Toronto Rehabilitation Institute, Toronto, ON, Canada

**Keywords:** cancer, cardiac rehabilitation (CR), Pulmonary rehabilitation (PR), prehabilitation, multi-disciplinary, exercise training

## Abstract

Exercise-based, multimodal rehabilitation programming similar to that used in the existing models of cardiac or pulmonary rehabilitation or prehabilitation is a holistic potential solution to address the range of physical, psychological, and existential (e.g., as their diagnosis relates to potential death) stressors associated with a cancer diagnosis and subsequent treatment. The purpose of this study was to systematically evaluate the structure and format of any type of exercise-based, multimodal rehabilitation programs used in individuals with cancer and the evidence base for their real-world effectiveness on metrics of physical (e.g., cardiorespiratory fitness, blood pressure) and psychological (e.g., health-related quality of life) health. Very few of the 33 included exercise-based, multimodal rehabilitation programs employed intervention components, education topics, and program support staff that were multi-disciplinary or cancer-specific. In particular, a greater emphasis on nutrition care, and the evaluation and management of psychosocial distress and CVD risk factors, with cancer-specific adaptations, would broaden and maximize the holistic health benefits of exercise-based rehabilitation. Despite these opportunities for improvement, exercise-based, multimodal rehabilitation programs utilized under real-world settings in individuals with cancer produced clinically meaningful and large effect sizes for cardiorespiratory fitness (VO_2_peak, ±2.9 mL/kg/min, 95% CI = 2.6 to 3.3) and 6-minute walk distance (+47 meters, 95% CI = 23 to 71), and medium effect sizes for various measures of cancer-specific, health-related quality of life. However, there were no changes to blood pressure, body mass index, or lung function. Overall, these findings suggest that exercise-based, multimodal rehabilitation is a real-world therapy that improves physical and psychological health among individuals with cancer, but the holistic health benefits of this intervention would likely be enhanced by addressing nutrition, psychosocial concerns, and risk factor management through education and counselling with consideration of the needs of an individual with cancer.

## Introduction

Aerobic and resistance exercise training are widely accepted as safe and effective interventions to improve cardiorespiratory fitness, physical function, quality of life, and acute and long-term cancer treatment-related side effects among individuals diagnosed with cancer (1). While exercise training provides numerous health benefits, a cancer diagnosis and subsequent treatment introduces a wide range of physical, psychological, and existential (e.g., as their diagnosis relates to potential death) stressors (2) that are unlikely to be adequately addressed by exercise alone. Exercise-based, multimodal rehabilitation programming similar to that offered in existing models of cardiac or pulmonary rehabilitation or prehabilitation prior to surgery includes aerobic exercise training as the cornerstone, in addition to education and/or counselling for nutritional and psychosocial concerns, and risk factor evaluation and management with the goal of promoting self-management strategies and fostering the adoption and maintenance of healthy lifestyle behaviors (3–5). A comprehensive, multi-disciplinary approach such as this is a holistic potential solution to address the myriad of sequelae associated with cancer.

One particularly detrimental long-term health consequence of cancer types with high cancer survival rates is a significant elevation in risk of cardiovascular disease (CVD)-related morbidity and mortality (6). The etiology of the increased CVD risk is proposed to arise from several factors including pre-existing CVD, pre-existing CVD risk factors (e.g., obesity, hypertension, and diabetes), cancer treatment-related cardiovascular toxicity, and pre-existing and/or treatment-related lifestyle toxicity (e.g., physical inactivity, unhealthy diet, tobacco use, increased psychosocial stress and weight gain) (7). Among heart disease patients, cardiac rehabilitation is an integral component of care as it improves cardiorespiratory fitness and quality of life and reduces CVD mortality (8). Cardiac rehabilitation is defined as “the provision of comprehensive long-term services involving medical evaluation, prescriptive exercise, cardiac risk factor modification, and education, counseling, and behavioral interventions” (9). In recognition of the potential benefit of a multimodal model of care for individuals with cancer, the American Heart Association (AHA) recently published a scientific statement recommending the use of a delivery model similar to cardiac rehabilitation, but with adaptations to address the needs of individuals with cancer, to mitigate CVD risk (10). The AHA statement also provided guidance for the adaptation of the structure of standard cardiac rehabilitation to encompass cancer-specific considerations and stated the need to establish the science base for cardiac rehabilitation in cancer populations to help establish reimbursement pathways (10).

Pulmonary rehabilitation and prehabilitation (i.e., prior to surgery) are similar multimodal models to cardiac rehabilitation that share the core components of exercise training, education, counselling, and risk factor evaluation and management (3, 4). Specifically, pulmonary rehabilitation can be defined as “a comprehensive intervention based on a thorough patient assessment followed by patient-tailored therapies that include, but are not limited to, exercise training, education, and behavior change, designed to improve the physical and psychological condition of people with chronic respiratory disease and to promote the long-term adherence to health-enhancing behaviors.” (11). Lung cancer is among the cancer types with the worst prognosis, owing in part to the high rate of surgical resection complications, which then limits treatment options for individuals with high risk factors (12). In this context, both prehabilitation prior to surgery and rehabilitation following surgery to optimize a patient's physical and psychological functioning has emerged as a potential solution.

With the first formal scientific statement from an international health organization advocating for the use of, and providing recommendations for exercise-based, multimodal rehabilitation programming to support individuals with cancer, a systematic evaluation of existing programs relative to these new guidelines and the evidence base for their impact on health outcomes is necessary to inform the optimization and adoption of exercise-based multimodal rehabilitation programs for individuals with cancer. In this context, the purpose of this study was to systematically evaluate exercise-based, multimodal rehabilitation programs such as cardiac or pulmonary rehabilitation or prehabilitation used in individuals with cancer in terms of their structure and health impacts. The first objective was to evaluate the setting, components, referral process, patient eligibility criteria, intake assessment of existing exercise-based multimodal programs in cancer populations relative to the AHA's cancer-specific recommendations to highlight gaps in care and areas for improvement for future programs. The second objective was to evaluate the real-world effectiveness of exercise-based, multimodal rehabilitation programs on physiological (i.e., cardiorespiratory fitness) and psychological (i.e., health-related quality of life) health among individuals with cancer by meta-analyses.

## Methods

This systematic review was conducted in accordance with the Preferred Reporting Items Systematic Reviews and Meta-Analyses (PRISMA) guidelines (13).

### Data Sources and Search Strategy

Six electronic databases were searched from inception to 16 April 2021: CINAHL Complete (EBSCOhost), Embase (Ovid), Emcare (Ovid), Medline (Ovid), PubMed (non-Medline), and Web of Science Core Collection. The search strategies were developed in collaboration with an Information Specialist [MP] and organized according to the relevant concepts of the PICO(S) framework encompassing Population/Problem, Intervention, Comparisons, Outcomes, and Study Design. Valid subject headings as appropriate for each database were utilized, as were free text terms pertinent to each topical concept.

The Population included adults with current or past cancer diagnosis. The Intervention focused on multimodal, exercise-based rehabilitation programs similar to the model used in cardiac or pulmonary rehabilitation or prehabilitation. A Comparator was not required. In order to keep the results as broad as possible to achieve the first objective of evaluating existing programs within cancer, no Outcomes were stipulated in the search strategy. Both retrospective and prospective (observational, single-arm or randomized) qualitative and quantitative Study Designs were included as this is a new research area. No date limits were applied, but the results were restricted to humans and studies published in English. The full Medline search strategy is shown in Supplementary Table 1.

### Study Selection

Included studies had to report separate data for adults with a history of any type of cancer diagnosis in English. The studies had to describe a structured, multimodal, exercise-based rehabilitation program that also included education to promote self-management strategies or mitigate CVD risk as the two core components of cardiac or pulmonary rehabilitation programs. Additionally, to capture real-world programs, the study had to include an explicit statement about the program being based on the cardiac or pulmonary rehabilitation model, being embedded in an existing rehabilitation program, or being the initiation of a new rehabilitation program. Studies that were purely based in a research setting were excluded. Original research studies ≥10 patients were included and reviews, case studies, editorials, commentaries, and research letters were excluded.

### Screening and Data Extraction

Titles/abstracts and full texts were reviewed independently for inclusion by two authors [JR, AE] using Covidence (Veritas Health Innovation, Melbourne, AUS; Available at http://www.covidence.org/). Discrepancies were reviewed and resolved by consensus. Data were extracted by one author and verified by a second [JR, AE, SS]. For each meta-analysis, the mean difference was calculated as the post-intervention outcome value minus the pre-intervention outcome value. Studies not reporting measures of variance (or only range) were not included in the meta-analysis. Study authors were contacted primarily for clarification on potential overlap of data between multiple manuscripts.

### Outcomes

The aspects of the programs evaluated were aligned with the AHA cardio-oncology rehabilitation recommendations which generally follow the structure for cardiac rehabilitation with adaptations for cancer-specific considerations. Specifically, the following components of the program structure were evaluated: the program setting, program components, referral process, patient eligibility criteria (including specifically CVD risk level), components of the baseline intake assessment, and patient enrollment and retention. The specific physiological, psychological, or patient-reported cancer-related (e.g., fatigue) outcome measures to be assessed in evaluation of the impact of rehabilitation programming were dictated by available data after the search was completed. The expected outcomes of interest were cardiorespiratory fitness (peak volume of oxygen consumption, VO_2_peak), exercise capacity (6-minute walk test distance), blood pressure, lipids, muscular strength, body weight, body mass index, quality of life, anxiety, and depression. Variables that were assessed at both time points (pre and post intervention) in at least three unique programs were analyzed via meta-analysis.

### Analyses

All evaluation criteria of the program structures are described by frequencies. Meta-analyses of the effect of the programs on available outcomes were performed using random effects models to allow for variation between studies, as the study samples and interventions included in this meta-analysis would be unlikely to have a common variance (14). Effect sizes were expressed as weighted mean difference (WMD) with 95% confidence intervals (CI). The standardized mean difference (SMD) is also provided to enable comparison and interpretation of the magnitude of effect size, where 0.2 = small, 0.5 = medium, and 0.8 = large (15). The risk of bias of an individual study was assessed by the leave-one-out sensitivity analysis approach. When a given study changed the statistical significance of the meta-analysis, results are provided for the analysis with and without that study. Interstudy heterogeneity was assessed using Cochrane Q statistic and quantified by I^2^ statistic (16).

Only the immediately post-intervention time point of evaluation was included as very few studies reported follow-up or interim time points. Subgroup analyses were planned a priori to evaluate the potential for differences in effect sizes for: (1) prehabilitation (defined as intervention taking place prior to treatment, often surgical treatment) vs. rehabilitation (defined as taking place after treatment but may overlap with a subsequent treatment); (2) studies that exclusively enrolled patients who were post-treatment (but may be receiving hormonal therapy) versus studies that included some (or all) patients on active treatment (with surgery, chemotherapy, or radiation) due to the potential for cancer treatment to reduce the effect size; (3) retrospective vs. prospective study design; and (4) common cancer types. The availability of studies reporting the same outcome in the same cancer type was too low to perform this subgroup analysis. An additional sub-group analysis was planned *post-hoc* to evaluate the effects of prehabilitation on lung cancer surgical outcomes based on the number of included studies that reported these clinically relevant outcomes. For all other subgroup analyses, only those with two or more programs per group available are reported. Due to the small program number within subgroups, within-group estimates of tau-squared were pooled for all subgroup analyses and random effects weights were used within subgroups.

## Results

### Study Selection

**Figure 1:**
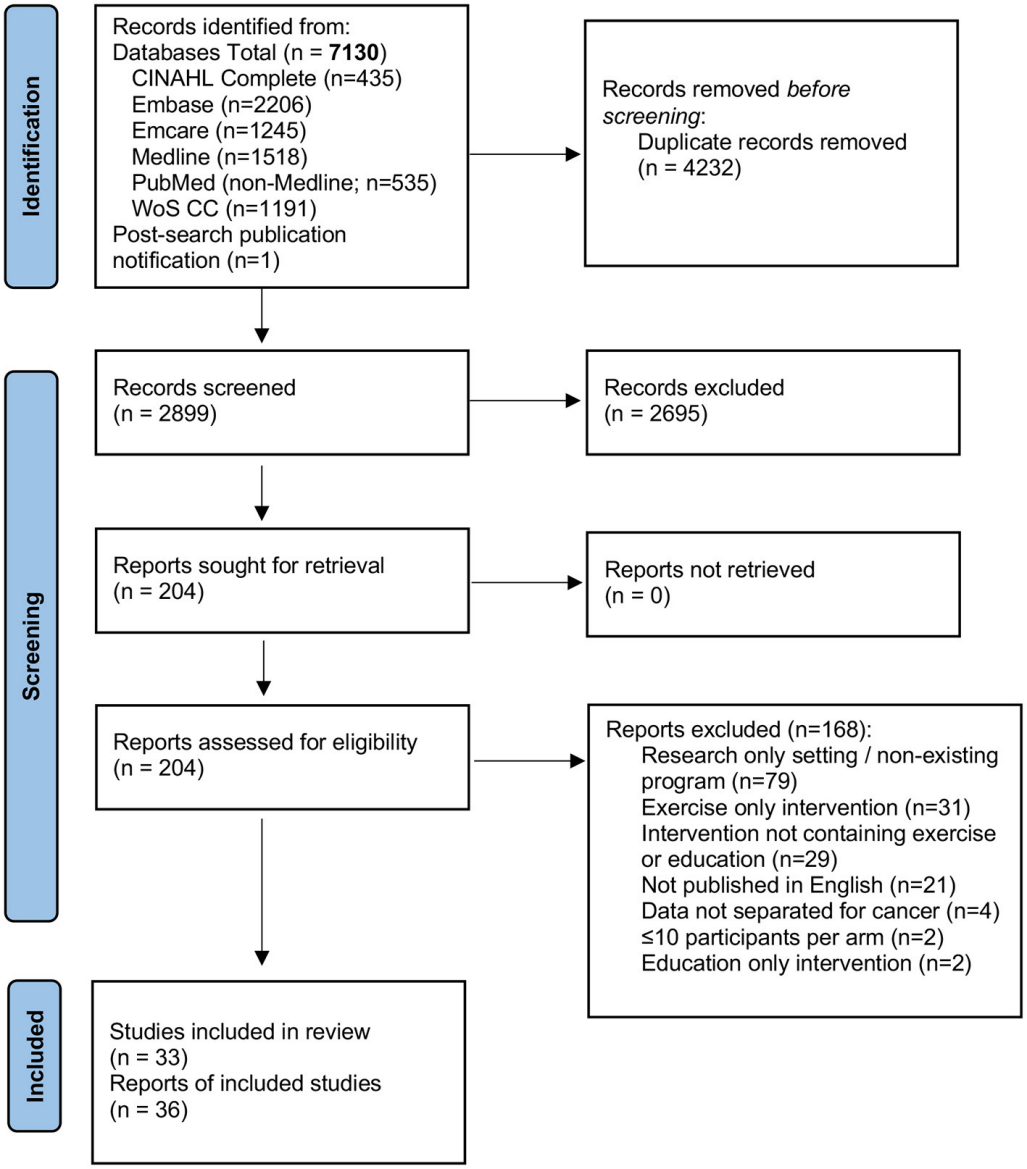
PRISMA flow diagram.

A total of 7,130 citations, or 3,749 original studies after removal of duplicates were retrieved with the search strategy. The full text of 203 articles was reviewed to select 35 manuscripts, while one additional manuscript that was published ~6 weeks after our search was completed was identified by the study team for a total of 36 manuscripts (17–52). Figure 1 shows the PRISMA diagram with exclusion reasons. In total these 36 manuscripts described 33 unique programs; three programs were described in 2–3 different manuscripts (26, 27, 32, 33, 38, 39, 47, 52), two manuscripts described 2 or 3 unique programs (22, 31).

### Evaluation of Programs Relative to Cardio-Oncology Rehabilitation Recommendations

#### Program Setting

The wide majority (*n* = 29, 88%) of programs were delivered in a clinical or hospital-based setting, while two (6%) were delivered in a community-based setting, and two (6%) were home-based (Supplementary Table 2).

#### Program Components

As per our inclusion criterion, all programs included exercise training and education. Within the exercise prescriptions (described in Supplementary Table 3), the majority (*n* = 24, 73%) included both aerobic and resistance exercise, while five (15%) and three (9%) included only aerobic or resistance, respectively, and one did not describe the exercise prescription. For those including aerobic exercise, the majority prescribed frequency as 2–3 times per week, at a moderate-to-vigorous intensity for a wide range of duration from 20 to 120 minutes. For those that specified an aerobic exercise mode, walking and cycle ergometry were the most common. The majority of the studies did not describe the details of the resistance exercise prescriptions beyond frequency, which ranged from once per week to daily. The prescribed resistance exercise intensity was infrequently described, but 8 (24%) programs explicitly stated that n-repetition-maximum testing performed at baseline was used as the basis of the prescription. One-third (*n* = 12, 36%) of programs prescribed unsupervised exercise to be performed at home in addition to supervised exercise. Only 5 (15%) studies explicitly stated the prescription of flexibility exercises.

Education topics included nutrition (*n* = 24, 73%), psychosocial concerns or stress management (*n* = 21, 64%), physical activity (*n* = 18, 55%), cancer-specific topics (e.g., lymphedema, cancer pathology, medications) (*n* = 10, 30%) and weight management (*n* = 2, 6%) (Supplementary Table 3). Ten (30%) studies reported performing goal setting within these education sessions.

Beyond exercise training and education, other core components of rehabilitation were not common (Supplementary Table 3). Dietary interventions such as cooking classes, supplementation, or a nutrition plan were described in five (15%) programs. Psychosocial interventions such as counselling were described in three (9%) programs. Tobacco cessation was provided in 10 (30%) programs, but half of these were in programs specifically for lung cancer. In terms of CVD factor management, just four (12%) programs reported both assessing and managing hypertension, dyslipidemia, or diabetes. Four (12%) programs reported referrals to other healthcare professionals (e.g., occupational, speech, or massage therapists) as a program component.

#### Referral Process

The most common referral source was the treating health care provider (n = 26, 79%; Supplementary Table 2). Only one study utilized self-referral, and the remainder allowed either self or provider referral (*n* = 5, 15%) or did not report (or imply) the source (*n* = 1, 3%).

#### Patient Eligibility

Seven (21%) programs allowed enrollment of any type of cancer and the most common type-specific programs were for lung cancer (*n* = 8, 24%) and breast cancer (*n* = 8, 24%; Supplementary Table 2). The majority (*n* = 23, 70%) of programs allowed enrollment of individuals diagnosed with any stage of cancer, while 10 (30%) only enrolled individuals with early-stage cancer, and none were specific to advanced cancer. Eligibility with respect to the timing of treatment was specified as during active treatment in 2 (6%), exclusively post-treatment in 9 (27%), at any time of the cancer trajectory in 4 (12%) and not specified in the remainder of programs. Eight (24%) programs were prehabilitation programs, with most being initiated in the pre-operative treatment window. Only one (3%) program described the presence or identification of CVD, cardiotoxicity, cardiac symptoms, or high CVD risk as a program inclusion criterion or requirement for referral. No studies described the requirement for receipt of cardiotoxic treatments such as high-dose anthracyclines or left-sided radiation for inclusion in the program. Five (15%) studies required poor pulmonary function, physical function, or exercise capacity among lung cancer patients for program inclusion (Supplementary Table 2).

#### Baseline Assessment

Cancer-specific, cardiovascular-specific, or general medical history were reported as occurring at the baseline assessment in 22 (67%) programs (Supplementary Table 3). Blood work was only explicitly stated as being required or reviewed in two (6%) programs and it was for cardiometabolic markers. The most commonly used subjective psychosocial assessments were quality of life (*n* = 17, 52%) and anxiety or depression (*n* = 13, 39%). Self-reported lifestyle behaviors were infrequently assessed, with 11 (33%) assessing physical activity, 6 (18%) assessing dietary practices, and 17 (52%) assessing tobacco use. Two (6%) programs objectively assessed baseline physical activity using accelerometers. The most commonly assessed cancer-specific condition was cancer-related fatigue (*n* = 10, 30%), while self-reported symptoms, lymphedema, and previous cardiac failure were each assessed in one study. Ten (30%) studies reported assessing basic anthropometrics (height, weight, body mass index), and three (9%) also assessed waist circumference. Blood pressure was reported as being assessed in 3 (9%) programs, while cardiorespiratory fitness (VO_2_peak) or exercise capacity (6-min walk test) was assessed in 11 (33%) and 9 (27%), respectively.

#### Program Staff

Program staff was generally poorly described in most studies (Supplementary Table 3), other than inclusion of an exercise professional, which was reported in 24 (73%) studies. Among these studies, 12 (50%) reported that the exercise staff was a physiotherapist, 2 (8%) reported a certified exercise professional, and the remaining did not specify. Nine (27%) studies included a physician, and of these, four (44%) reported that the physician had cancer-specific training or experience. Eleven (33%) studies reported including a registered dietitian or nutritionist, 13 (39%) reported including a mental health professional, and 14 (42%) reported including an oncology care provider. Only one study reported that the exercise, nutrition, or mental health professionals had oncology-specific training.

### Meta-Analysis

#### 6-Minute Walk Test Distance

**Figure 2:**
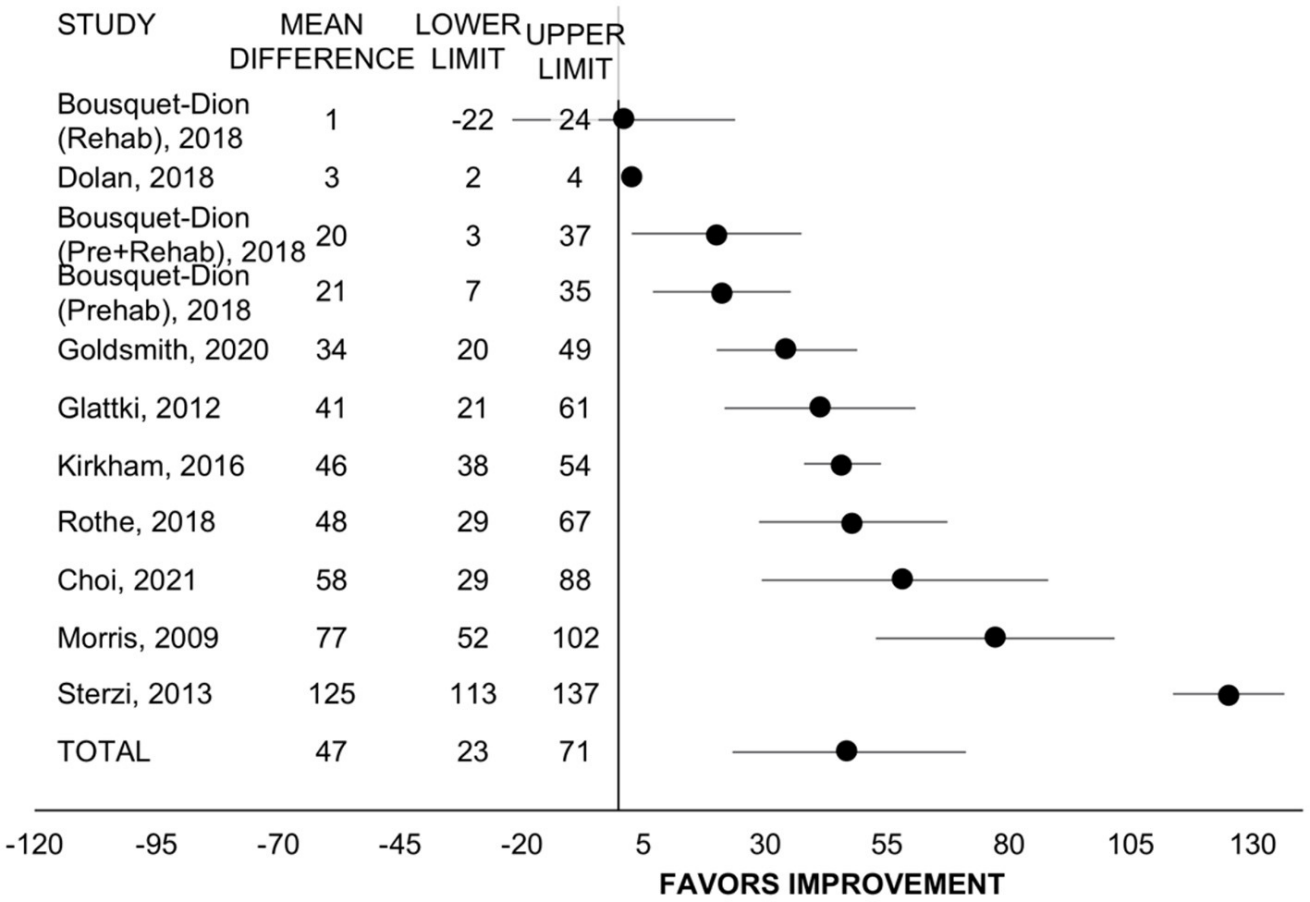
Forest plot displaying mean difference and 95% CIs for the impact of pre/rehabilitation on 6-minute walk test distance (meters).

Eight studies with *n* = 680 participants among 10 different programs evaluated change in six-minute walk test distance (22, 24, 29, 30, 37, 42, 45, 46). Including all programs, pre/rehabilitation improved six-minute walk distance by 47 meters (95% CI = 23 to 71, SMD = 0.78, *p* < 0.001, I^2^ = 96%; Figure 2). When comparing program type, the improvement in six-minute walk distance was only significant for rehab (*n* = 7, WMD = 57 m, 95% CI = 29 to 85, *p* < 0.001, I^2^ = 96%) (20, 22, 27, 35, 40, 43, 44) and not for prehab (*n* = 3, WMD = 25 m, 95% CI = −16 to 67, *p* = 0.236, I^2^ = 11%) (20, 28) but was not statistically different between types (*p* = 0.211). For treatment timing, the improvement was significant only among programs with participants exclusively post-treatment (*n* = 7, WMD = 50 m, 95% CI = 18 to 81, *p* = 0.002, I^2 =^ 97%) (22, 24, 29, 30, 42, 45) and was only a trend among programs that allowed patients on active treatment with surgery, chemotherapy, or radiation (*n* = 3, WMD = 41 m, 95% CI = −8 to 90, *p* = 0.100, I^2^ = 76%) (22, 24, 37) but did not differ between subgroups (*p* = 0.765). Retrospective studies had larger improvements in six-minute walk distance (*n* = 5, WMD = 70 m, 95% CI = 39 to 101, *p* < 0.001, I^2^ = 97%) (24, 29, 37, 42, 46) than prospective (*n* = 5, WMD = 25 m, 95% CI = −5 to 56, *p* = 0.107, I^2^ = 66%) (*p* = 0.042 between groups).

#### VO_2_peak

**Figure 3:**
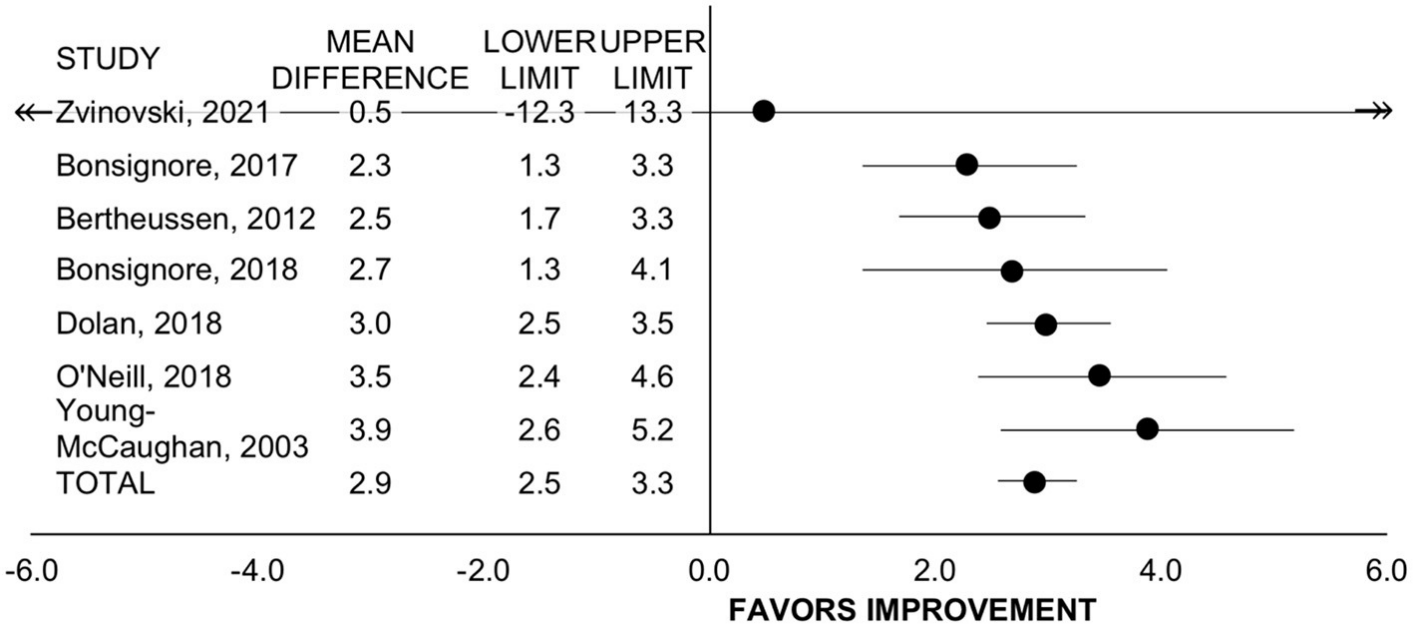
Forest plot displaying mean difference and 95% CIs for the impact of rehabilitation on VO_2_peak (mL/kg/min).

Seven studies with *n* = 373 participants evaluated change in VO_2_peak via cardiopulmonary gas analysis (*n* = 4) (20, 27, 43, 52) or estimation from workload (*n* = 3) (19, 49, 51). Of these studies, all employed rehabilitation programs. Including all studies, rehabilitation improved VO_2_peak by 2.9 mL/kg/min (95% CI = 2.6 to 3.3, SMD = 0.75, *p* <0.001, I^2^ = 0%; Figure 3). For treatment timing, the improvement was significant for programs that allowed patients on active treatment (*n* = 2, WMD = 3.1 mL/kg/min, 95% CI = 2.6 to 3.6, *p* < 0.001, I^2^ = 33%) (27, 49) and for programs including patients exclusively post-treatment (*n* = 5, WMD= 2.7 mL/kg/min, 95% CI = 2.2 to 3.2, *p* < 0.001, I^2^ = 0%) (19, 20, 43, 51, 52) and did not differ between subgroups (*p* = 0.257). The change in VO_2_peak did not differ between studies with retrospective vs. prospective design (*p* = 0.578).

#### Cancer-Specific, Health-Related Quality of Life

Eleven studies reported change in health-related quality of life measures that were cancer-specific among 12 programs and with several studies reporting more than one measure of quality of life (19, 27, 31, 34, 36–39, 43, 44, 47, 51). Of these, *n* = 6 used the European Organisation for Research and Treatment of Cancer quality of life questionnaire (EORTC-QLQ)-C30 in *n* = 2008 participants, *n* = 5 used the Functional Assessment of Cancer Therapy (FACT)-General in *n* = 300 participants, *n* = 4 used the FACT-Breast total in *n* = 206 participants, *n* = 4 used the FACT-Fatigue in *n* = 1410 participants. All studies employed rehabilitation programming.

**Figure 4:**
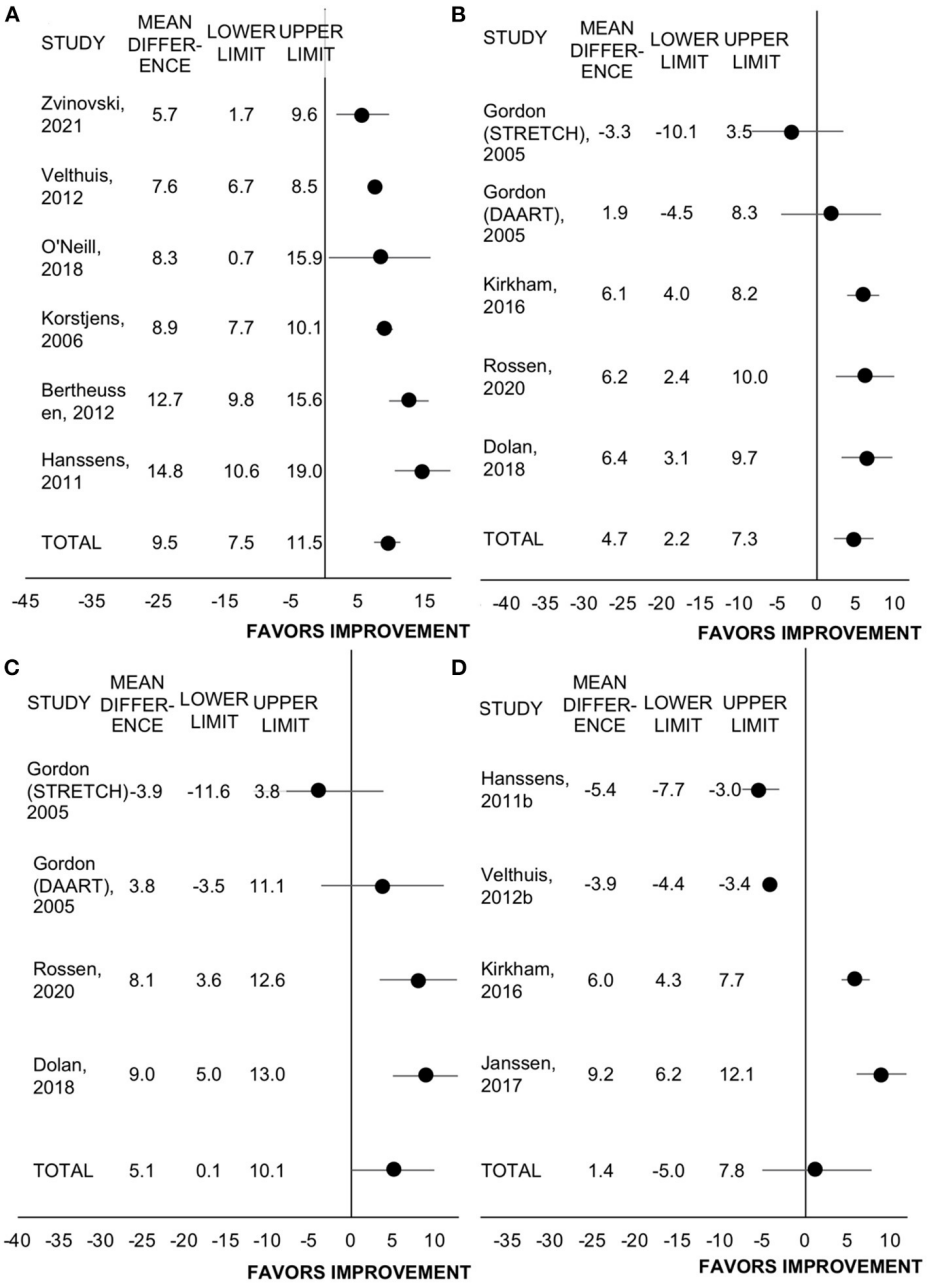
Forest plot displaying mean difference and 95% CIs for the impact of rehabilitation on measures of health-related quality of life: **(A)** EORTC-QLQ-C30; **(B)** FACT-General; **(C)** FACT-Breast; **(D)** FACT-Fatigue.

All studies utilizing the EORTC-QLQ-C30 included patients who had completed treatment, while all studies utilizing the FACT included patients on active treatment. Rehabilitation improved the EORTC-QLQ-C30 by 9.5 points (95% CI = 7.5 to 11.5, SMD = 0.30, *p* < 0.001, I^2^ = 78%; Figure 4). Rehabilitation also improved FACT-General by 4.7 points (95% CI = 2.2 to 7.3, SMD = 0.62, *p* < 0.001, I^2^ = 52%; Figure 4). The effect size of these two general quality of life questionnaires combined was medium (SMD = 0.49, 95% CI = 0.37 to 0.62, *p* < 0.001, I^2^ = 77%).

Rehabilitation improved the FACT-Breast total score by 5.1 points (95% CI = 0.1 to 10.1, SMD = 0.26, *p* = 0.044, I^2^ = 98%). However, in the leave-one-out sensitivity analyses, with the removal of any of the studies by Dolan et al., Gordon et al. (DAART program only), or Rossen et al., the change in FACT-Breast was no longer significant (*p* ≥ 0.10). Including all studies, rehabilitation did not change FACT-Fatigue score (WMD = 1.4, 95% CI = −5.0 to 7.8, SMD = 0.11, *p* = 0.667, I^2^ = 68%). The FACT-Fatigue effect size did not differ between retrospective and prospective studies (*n* = 2 each, *p* = 0.916).

#### Anthropometrics

Six studies with *n* = 226 participants reported body mass index (20, 21, 37, 43, 51, 52) and three with *n* = 295 participants reported body mass (27, 37, 43) before and after the program. Prehab/rehab did not change body mass index (WMD = 0 kg/m^2^, 95% CI = −0.3 to 0.4, SMD = 0.02, *p* = 0.777, I^2^ = 0%) and this did not differ by treatment timing (*p* = 0.728) or study design (*p* = 0.576). Rehabilitation did not change body mass (WMD = −0.2 kg, 95% CI = −0.9 to 0.6, SMD = −0.05, *p* = 0.675, I^2^ = 62%).

#### Spirometry

Four studies with *n* = 145 participants reported forced expiratory volume in one second (FEV_1_) (24, 29, 41, 46) and three with *n* = 125 participants reported forced vital capacity (FVC) (24, 29, 46). Pre/rehabilitation did not impact FEV_1_ (WMD = 4.6 % predicted, 95% CI = −5.4 to 14.5, SMD = 0.69, *p* = 0.369, I^2^ = 98%) or FVC (WMD = 1.0 % predicted, 95% CI = −4.4 to 6.4, SMD = 0.24, *p* = 0.719, I^2^ = 89%). However, sensitivity analysis showed that the removal of the study by Sterzi et al. resulted in a significant improvement in FEV_1_ (WMD = 9.0% predicted, 95% CI = 2.1 to 15.8, *p* = 0.011, I^2^ = 92%) and FVC (WMD = 3.9% predicted, 95% CI = 2.1 to 5.8, *p* < 0.001, I^2^ = 0%).

#### Blood Pressure

Four studies with *n* = 183 participants reported blood pressure (20, 37, 51, 52) and it was not affected by rehabilitation (systolic: WMD = −1.5 mmHg, 95% CI = −3.5 to 0.5, SMD = −0.11, *p* = 0.135, I^2^ = 0%; diastolic: WMD = −0.3 mmHg, 95% CI = −1.6 to 1.1, SMD = −0.01, *p* = 0.722, I^2^ = 29%).

There were not three or more unique programs that measured fasting glucose, hemoglobin A1c, lipid profile, smoking cessation success rates, nutritional intake, or depression or anxiety (with the same measure) to allow meta-analysis.

### Post hoc Analysis of Prehabilitation for Surgical Resection of Lung Cancer

The only programs that reported surgical outcomes were five prehabilitation programs implemented prior to surgical resection for lung cancer (21, 23, 30, 41, 50). Four of these studies compared post-operative outcomes, including complications (e.g., pneumonia, infections), and length of hospital stay between patients who participated in prehabilitation to patients that did not participate (21, 23, 30, 50) but could not be combined by meta-analysis due to variability in outcome reporting formats. All four studies reported a significant reduction or trend to significance for incidence of various post-operative complications and post-operative hospital stay length among participants in the prehabilitation program (21, 23, 30, 50). Two studies reported significant improvements in FEV_1_ and exercise capacity following the short prehabilitation program (typically lasting 1–4 weeks) (23, 41). Importantly, these health effects translated to a significant increase in the proportion of participants able to receive surgery (30% vs. 69%) in one program (30) and reduced post-operative health care costs in two programs (21, 23).

## Discussion

Through the systematic review, evaluation, and meta-analyses of exercise-based multimodal rehabilitation programs such as cardiac or pulmonary rehabilitation or prehabilitation utilized in individuals diagnosed with cancer, this review summarizes the available evidence base for the effectiveness of these interventions and is a first step towards informing the optimal components, staff, referral process, and setting of future programs. Few of the published exercise-based, multimodal, rehabilitation programs utilized in individuals with cancer have adhered to the AHA's cancer-adapted recommendations for rehabilitation, especially with respect to inclusion of multi-disciplinary and/or cancer-specific interventions and program support staff. However, despite the lack of cancer considerations and holistic approach to rehabilitation employed by the published programs to-date, there were statistically and clinically significant improvements in exercise capacity, cardiorespiratory fitness, and health-related quality of life. Based on these findings, we have identified a number of areas of the program structure with room for improvement to better accommodate the unique needs of individuals with cancer to maximize the size and duration of health benefits of an exercise-based, multimodal rehabilitation approach.

### Program Components

Cardiorespiratory fitness is one of the strongest independent predictors of all-cause, cancer-related, and CVD-related mortality in cancer survivors (53). Exercise training is the most effective intervention for improving cardiorespiratory fitness and as such should be a cornerstone of multi-modal programs for individuals diagnosed with cancer, similar to contemporary cardiac and pulmonary rehabilitation (3, 4, 10). An important finding from the current meta-analysis is that real-world, exercise-based, multimodal rehabilitation resulted in the same effect size of improvement in VO_2_peak, the gold standard measure of cardiorespiratory fitness, as compared with rigorously controlled randomized controlled trials of exercise training in individuals with cancer reported in a meta-analysis (2.9 vs. 2.8 mL/kg/min) (54). The six-minute walk test is another reliable measure of exercise capacity that is correlated with cardiorespiratory fitness and also predicts cancer outcomes (55–57). Our meta-analysis also identified a clinically meaningful mean improvement of 47 m in six-minute walk distance with real-world rehabilitation which exceeds the threshold of 42 m identified as a clinically importance change among individuals with lung cancer (58).

While exercise training provides numerous health benefits, a cancer diagnosis and subsequent treatment introduces a wide range of physical, psychological, and existential stressors (2) that are unlikely to be adequately addressed by exercise alone. In particular, nutrition is a core component of traditional cardiac or pulmonary rehabilitation programming that is of high relevance for cancer populations but was inadequately addressed in most programs in this study. While 73% of programs reported providing education on nutritional topics, this may have been limited to a single informational session. Only 14% of programs described any type of nutritional intervention where dietary counselling or planning was provided. Malnutrition is common among individuals diagnosed with cancer due to the combined effects of the tumor itself, anticancer treatments, and poor dietary habits, and its presence contributes to reduced quality of life, increased treatment toxicity, and death independent of cancer (59). The 2021 European Society for Clinical Nutrition and Metabolism (ESPEN) guidelines on nutrition in cancer patients reported that despite the robust evidence base for the crucial role of nutrition as a critical component of multimodal cancer care, it is largely unrecognized, underestimated, and undertreated in practice (59). Among the ESPEN's 43 recommendations for nutritional and metabolic management of patients with cancer, is the recommendation for use of nutritional interventions that provide dietary advice, addressing treatment and nutritional impact symptoms that impair food intake, and offering supplements. These aspects of nutrition support are within the scope of current practice of cardiac rehabilitation program registered dietitians. A greater emphasis on nutrition care as a component of rehabilitation is likely to compound health benefits received from exercise for individuals with cancer. Furthermore, in cancer survivors who are at greater risk for obesity and metabolic impairments (e.g., glucose intolerance) than for malnutrition (e.g., early-stage breast cancer, endometrial cancer), nutritional interventions are needed to normalize glycemic control, induce fat mass loss, and other associated markers of CVD risk. The optimal intervention for inducing these benefits would be one that takes advantage of the synergies in a multimodal diet and exercise intervention.

Psychosocial distress is extremely prevalent among individuals with cancer, especially within the first year after a diagnosis but this may persist long after completion of primary treatment (2). For example, within the first year of a breast cancer diagnosis, ~50% of women experience depression, anxiety, or both (60). Depression can increase the risk of developing other comorbidities such as CVD, increases CVD mortality risk, and can reduce adherence to rehabilitation if not addressed (61, 62). The psychological health of individuals with cancer is determined by more than simply the absence of distress, but also by the presence of positive psychological responses such as self-esteem, life appreciation and meaning, spirituality, and feelings of peacefulness and purposefulness (2). Screening for and then treating psychosocial distress through education, goal setting, individual or group counselling within the rehabilitation setting can facilitate these positive psychological responses and in turn, reduce the risk of onset of new comorbidities and death. Depression may also reduce adherence and completion rates to multi-modal programming such as cardiac rehabilitation, therefore addressing psychosocial concerns may increase engagement with these potentially life-saving services (63). Only 9% of the included programs in this study reported including psychosocial interventions of any kind, which represents an actionable programmatic change that could significantly enhance the effects of multimodal rehabilitation on the well-being of individuals with cancer.

CVD is a primary competing risk of death for individuals diagnosed with numerous cancer types, especially among those with higher cancer survival rates (6). The risk of CVD-related death is elevated at all points in the survivorship trajectory after a cancer diagnosis when compared to the general population (6). The assessment and management of modifiable CVD risk factors (e.g., blood pressure, glucose, lipids, tobacco use) is an integral component of cardiac rehabilitation but was rarely employed in the programs in the current study. This finding suggests that the majority of rehabilitation programs used in individuals with cancer to-date have not had a focus on mitigation of CVD risk, which represents a missed opportunity to enhance the overall health profile of individuals with cancer by targeting a primary competing risk of death. Furthermore, a shift toward a focus on CVD risk reduction in rehabilitation for cancer types where CVD is a prevalent competing risk may provide a viable avenue for reimbursement from third-party payers based on the precedent set by cardiac rehabilitation. Although offering multi-modal programming including exercise, psychosocial, nutritional, and CVD risk factor modification support is optimal, this might not be viable in all centers, especially in low resource settings. Suggestions for adapting cardiac rehabilitation for low resource settings include a menu-based and flexible implementation of recommendations as possible, use of non-physician-led interventions for low risk patients, or delivery through the community, home, internet/mobile technology, or within primary care settings (64).

### Referral Process and Setting

#### Referral Timing

The AHA cardio-oncology rehabilitation guidelines suggest that referral to rehabilitation should be driven by prior cardiotoxic exposures and current symptoms, rather than considerations of treatment timing (e.g., before, during, or after) relative to treatment (10). Some components of rehabilitation, especially exercise, introduce additional challenges and considerations when performed during active treatment where patients have time constraints due to appointments and tests, are immunocompromised, and experiencing symptoms such as nausea, emesis, fatigue, and depression. However, exercise training during active treatment has been shown to reduce treatment-related toxicities and potentially impact medical outcomes such as reducing hospitalizations, chemotherapy complications, and reduced dose intensity (66). The results from the current meta-analysis support the implementation of rehabilitation prior to, during, or after primary treatment completion. Our subgroup comparisons of programs that included vs. excluded patients on active treatment and prehabilitation vs. rehabilitation showed that effect size for cardiorespiratory fitness, exercise capacity and quality of life did not differ and was clinically meaningful regardless of timing. This finding suggests that referral timing can be based on individual patient preference or ability. However, our finding that prehabilitation improved surgical outcomes suggests that intervention timing may be important in this context.

#### Referral Source

The wide majority of programs enrolled patients following referral by a treating health care provider, which was not often an oncologist or cardiologist. A referral by a health care provider may be a requirement for reimbursement of centre-based rehabilitation costs by a third-party payer. Referral rates to cardiac and pulmonary rehabilitation are alarmingly low, with only 28% of patients undergoing cardiac catheterization and less than 35% of eligible patients being referred, respectively (67, 68). Referral of eligible patients with cancer to rehabilitation will likely face similar challenges. The streamlining or automation of referrals from specialists (related to cardiac or pulmonary care), the oncology care team, and primary care providers, and endorsement from the treating physician are a few approaches that could be considered to enhance referrals and uptake of multimodal rehabilitation among individuals with cancer.

#### Program Setting

Only three (9%) programs in this study took place outside of a clinical setting such as in the community or at home. A recent scoping review of 31 unique programs offering supervised exercise in a community-based setting reported that it was safe and improved health-related quality of life for an individual with cancer (69). Consideration of patient preference, safety, and efficacy is required to determine appropriateness of center-based, home-based or community-based programming for cancer survivors. The AHA cardio-oncology rehabilitation guidelines recommend the use of centre-based rehabilitation for individuals with higher cardiac risk features (e.g., history of CVD, cardiac symptoms, receipt of cardiotoxic treatments, and presence of CVD risk factors), whereas those without these risk factors are appropriate candidates for community-based programming. Despite most of the programs being clinical centre-based in this study, only one study had the requirement for high cardiac risk features for enrollment and in fact, a number of programs excluded patients with these factors. It is not feasible or needed for all individuals with cancer to attend rehabilitation. CVD risk stratification is a viable method to identify and target those in greatest need of intervention.

### Program Staff

Oncology-specific certification is available for exercise, nutrition, and mental health professionals (69–72), but only one study reported that their staff had this training. While adequate knowledge of cancer diagnoses and treatments is critical for the safe and effective provision of support for individuals with a history of cancer, the integration of oncology rehabilitation into existing cardiac or pulmonary rehabilitation programs where resources are already limited, may not allow for the additional cost and time associated with additional training of the program staff. It may be more feasible for the cardiac/pulmonary rehabilitation programs to partner with the local cancer treatment centers to share personnel or receive informal training.

### Program Funding

A reimbursement strategy is currently unavailable for individuals with cancer participating in cardiac rehabilitation, highlighting the need to demonstrate the effectiveness of these programs in this population (10). In cardiac populations, cardiac rehabilitation has been found to be cost-effective relative to no cardiac rehabilitation, but the most cost-effective delivery model is unclear (65, 73). In cancer survivors, exercise physiologist- or physiotherapist-led physical activity programs have been shown to be more cost-effective compared to home-based self-management programs (74). As such, the cardiac rehabilitation model may be a cost-effective intervention to support cancer survivors. Implementing cancer rehabilitation programs using existing cardiac or pulmonary rehabilitation infrastructure may be a viable solution to reduce costs for utilization of these programs by cancer survivors. However, among the general cardiac rehabilitation landscape in Canada, approximately 200,000 more cardiac spots are needed per year to treat patients with ischemic heart disease alone, not including other patient population such as those living with atrial fibrillation, heart failure, and valvular disease (75). Therefore, funding constraints may limit enrollment of large numbers of cancer survivors directly into existing cardiac/pulmonary rehabilitation programs. Dedicated funding, resources, and staff are likely needed to enhance capacity for individuals with cancer to participate in exercise-based multimodal rehabilitation programming.

### Limitations

The results of this meta-analysis are limited by the lack of a control (non-rehabilitation) comparison group, small sample sizes, heterogeneity in results, and inability to perform subgroup analysis by cancer type. Within each evaluated outcome measure, there were too few studies including patients with a given cancer diagnosis type to allow for subgroup comparisons. As such, some analyses had moderate-to-high heterogeneity as indicated by I^2^, which indicates genuine differences in results between studies which could be related to grouping various cancer types, stages, and timing relative to treatment together. However, random effects models were used because heterogeneity was expected, and they provide estimates of the average intervention effect.

## Conclusion

This systematic evaluation of the structure of 33 existing exercise-based multimodal rehabilitation programs utilized in individuals with cancer to-date identified that the program components, education topics, and staff were not often multi-disciplinary and/or cancer-specific. In particular, a greater emphasis on nutrition, and evaluation and management of psychosocial distress and CVD risk factors, similar to the program components often offered in traditional cardiac rehabilitation but with cancer-specific adaptations, would provide enhanced holistic health benefits. Further, the wide majority of rehabilitation programs used in individuals with cancer to-date have not required elevated CVD risk (including receipt of cardiotoxic treatments) as an enrollment criterion nor have their program intake assessment or components had a focus on evaluation and mitigation of CVD risk. A shift toward a focus on CVD risk reduction in rehabilitation for cancer types where CVD is a prevalent competing risk will enhance the potential health impact of these programs. This study also found that cardiac rehabilitation, pulmonary rehabilitation, prehabilitation or other exercise-based multimodal rehabilitation programs utilized under real-world settings in individuals with cancer produced clinically meaningful and large effect sizes for cardiorespiratory fitness and 6-minute walk distance, and medium effect sizes for cancer-specific, health-related quality of life. Overall, these findings suggest that a model of exercise-based multimodal rehabilitation that addresses risk factors and nutritional and psychosocial concerns, and risk factors through education and counselling with consideration of the needs of individuals with cancer could be a holistic solution to address the range of physical, psychological, and existential stressors associated with a cancer diagnosis and subsequent treatment.

## Data Availability Statement

The raw data supporting the conclusions of this article will be made available by the authors, without undue reservation.

## Author Contributions

MP developed search strategy for the review and assisted with methods description. JR and AE completed article screening. JR, AE, and SS completed data extraction. AE created PRISMA diagram. JR and SS created tables. AK performed meta-analysis. AK, AB, and JR prepared the manuscript. PO is an expert contributor and assisted with manuscript completion. All authors contributed to the article and approved the submitted version.

## Conflict of Interest

The authors declare that the research was conducted in the absence of any commercial or financial relationships that could be construed as a potential conflict of interest.

## Publisher's Note

All claims expressed in this article are solely those of the authors and do not necessarily represent those of their affiliated organizations, or those of the publisher, the editors and the reviewers. Any product that may be evaluated in this article, or claim that may be made by its manufacturer, is not guaranteed or endorsed by the publisher.
