# Supplementary material for: Evaluation of the Structure and Health Impacts of Exercise-Based Cardiac and Pulmonary Rehabilitation and Prehabilitation for Individuals With Cancer: A Systematic Review and Meta-Analysis
Source: Front Cardiovasc Med. 2021 Sep 22;8:739473. doi: 10.3389/fcvm.2021.739473 (PMC8494200; doi:10.3389/fcvm.2021.739473)
Supplement: Supplementary file 1 [file Data_Sheet_1.PDF]

Supplemental Material Table 1: Ovid MEDLINE Search Strategy (<1946 to April 16, 2021)

1 [Population: Cancer & Survivors]
2 Cancer Survivors/ (4862)
3 exp neoplasms/ (3444343)
4 Hematopoietic Stem Cell Transplantation/ (43915)
5 (cancer* or neoplasm* or malignanc* or neoplasia* or tumor* or tumour*).tw,kw. (3139633)
6 or/2-5 (4425264)
7 [Intervention: Exercise-based Multi-Modal Rehab/Prehab Programs]
8 Cardiac Rehabilitation/ (2714)
9 Preoperative Exercise/ (52)
10 exp Exercise/ and (multimodal or multi-modal or multimodel or multi-model or rehab* or prehab*).tw,kw. (10011)
11 exp Exercise Therapy/ and (multimodal or multi-modal or multimodel or multi-model or rehab* or prehab*).tw,kw. (11875)
12 ((rehab* or prehab*) adj3 (cardiac or cardio* or CR-based or exercis* or pulmonary)).tw,kw. (16470)
13 ((exercis* or rehab* or prehab* or training) adj5 (multimodal or multi-modal or multimodel or multi-model)).tw,kw. (1191)
14 ((exercis* or resistance training or strength training or aerobic training or cardiovascular training) adj3 (rehab* or prehab*)).tw,kw. (6097)
15 or/8-14 (31045)
16 6 and 15 (1530)
17 limit 16 to "humans only (removes records about animals)" (1518)

Supplemental Material Table 2: Study characteristics and patient eligibility

Publication	Study Design	Enrollment n; n (%) retention	Setting	Referral Source	Cancer Type	Rehab Timing Relative to T_x	Other Medical Inclusion Criteria
Baimas-George, 2020	RCT	10 enrolled; 10 (100%) retained	Clinical	Treating provider	All stages, hepato-pancreato-biliary	During neoadjuvant CT, 3-4 mo prior to S_x	N/A
Berglund, 1994	RCT	98 enrolled; 90 (92%) retained	NR	Treating provider	Any	Within 2 mo post CT or RT	N/A
Bertheussen, 2012	Prospective single-arm	163 enrolled; 134 (82%) retained	Clinical	Treating provider	Any	Completed primary T_x	KPS ≥70
Bonsignore, 2017; Dolan, 2018	Retrospective	Bonsignore: 29 enrolled; 19 (66%) retained; Dolan: 152 enrolled; 152 (100%) retained	Clinical	Treating provider	Early-stage, breast	Any	Bonsignore: Heart failure related to T_x Dolan: N/A
Bonsignore, 2018	Retrospective	39 enrolled; 27 (69%) retained	Clinical	Treating provider	Any stage, prostate	Any	Referred for CVD
Boujibar, 2017	Retrospective	19 enrolled; 19 (100%) retained	Clinical	Treating provider	Early-stage, NSCLC	Prior to S_x	VO_2peak ≤20 mL/min/kg
Bousquet-Dion, 2017 (Prehab)	RCT	41 enrolled; 41 (100%) retained	Clinical	Treating provider	Early-stage, colon or rectal	Prior to S_x	N/A
Bousquet-Dion, 2017 (Rehab)	RCT	32 enrolled; 26 (81%) retained	Clinical	Treating provider	Early-stage, colon or rectal	Immediately post S_x	N/A
Bousquet-Dion, 2017 (Pre+Rehab)	RCT	41 enrolled; 37 (90%) retained	Clinical	Treating provider	Early-stage, colon or rectal	Prior to S_x	N/A
Bradley, 2013	Prospective non-randomized	58 enrolled; 58 (100%) retained during prehab, 28 (48%) retained for rehab	Clinical	Treating provider	Any stage, lung	Prior to S_x	Fit for curative lung cancer S_x
Choi, 2021	Retrospective	15 enrolled; 11 (73%) retained	Clinical	Treating provider	Any stage, thoracic area	Concurrent to RT	N/A
Dennett, 2019	Prospective single-arm	N/A – qualitative assessment of program only	Clinical	Treating provider or self	Any	Any	N/A
Doganay, 2020; Halliday, 2020; Halliday, 2021	Prospective single-arm	Doganay: 39 enrolled; 39 (100%) retained Halliday 2020: 83 enrolled; 72 (87%) retained; Halliday 2021: 67 enrolled; 67 (100%) retained	Home	Treating provider or self	Any stage, esophago-gastric	Prior to T_x	N/A

Supplemental Material Table 2 continued: Study characteristics and patient eligibility

Publication	Study Design	Enrollment n; n (%) retention	Setting	Referral Source	Cancer Type	Rehab Timing Relative to T_x	Other Medical Inclusion Criteria
Gaskin, 1989; Gordon, 2005 (STRETCH)	Prospective single-arm	Gaskin: 114 enrolled; 47 (41%) retained Gordon: 31 enrolled; 28 (90%) retained	Clinical	Self	Early-stage, breast	≥8 wks post S_x	N/A
Glattki, 2012	Retrospective	47 enrolled; 47 (100%) retained	Clinical	Treating provider	Any stage, NSCLC	≥1 mo post T_x	N/A
Goldsmith, 2020	Prospective single-arm	216 enrolled; Unclear n retained	Clinical	Treating provider	Any stage, lung	Prior to T_x	dyspnoea or WHO performance status >1, age >70 y or frailty index >3, reduced FEV_1 or DLCO
Gordon, 2005 (DAART)	Prospective single-arm	36 enrolled; 30 (83%) retained	Home	Treating provider	Early-stage, breast	3 wks post-diagnosis	N/A
Hanssens, 2011	Prospective single-arm	36 enrolled; 27 (75%) retained	Clinical	Treating provider	Any	Completed primary T_x	N/A
Heim, 2007	RCT	32 enrolled; 32 (100%) retained	Clinical	Treating provider	Any stage, breast	NR	≥4 on 0-10 fatigue scale
Janssen, 2017	Prospective single-arm	50 enrolled; 43 (86%) retained;	Clinical	Treating provider	Early-stage, NSCLC	6 wks post S_x	N/A
Kirkham, 2016	Retrospective	299 enrolled; 132 (44%) retained	Clinical	Treating provider or self	Any	During or after T_x	N/A
Korstjens, 2006; Korstjens, 2007; Velthuis, 2012	Prospective single-arm	Korstjens 2006: 658 enrolled; 658 (100%) retained Korstjens 2007: N/A – qualitative assessment of program only Velthuis: 1236 enrolled; 1078 (87%) retained	Clinical	Treating provider	Any	Completed primary T_x at least 2 mo prior	N/A
Kröz, 2017	RCT + prospective single-arm	54 enrolled; 41 (76%) retained	Clinical	Treating provider or self	Early-stage, breast	≥36 mo post primary T_x & before 45 mo post-diagnosis	chronic cancer-related fatigue for ≥6 mo
Marhic, 2018	Prospective single-arm	20 enrolled; 20 (100%) retained	Clinical	Treating provider	Early-stage, NSCLC	Prior to S_x	FEV_1 & VO_2peak <normal, high risk of cardiac complications, severe comorbidities, unfit for pulmonary resection

Supplemental Material Table 2 continued: Study characteristics and patient eligibility

Publication	Study Design	Enrollment n; n (%) retention	Setting	Referral Source	Cancer Type	Rehab Timing Relative to T_x	Other Medical Inclusion Criteria
Morris, 2009	Retrospective	30 enrolled; 30 (100%) retained	Clinical	Treating provider	Any	N/A	Chronic dyspnea, exercise intolerance or decreased functional status
O'Neill, 2018	RCT	21 enrolled; 21 (100%) retained	Clinical	Self	Early stage, esophageal, esophago-gastric, or gastric	Completed T_x between 6 mo & 5 y earlier	N/A
Rossen, 2020	Prospective single-arm	82 enrolled; 58 (71%) retained	Community	Treating provider	Any stage, breast	Any	N/A
Rothe, 2018	Prospective single-arm	45 enrolled; 30 (67%) retained	Clinical	Treating provider	Any stage, lymphoma	6 wks post HSCT	N/A
Sterzi, 2013	Retrospective	110 enrolled; 110 (100%) retained	Clinical	Treating provider	Any stage, NSCLC	Post S_x	N/A
Wu, 2021	Prospective single-arm	24 enrolled; 24 (100%) retained	Clinical	Treating provider	Any stage, breast	Within 18 mo of T_x	N/A
Young-McCaughan, 2003	Prospective single-arm	62 enrolled; 46 (74%) retained	Clinical	Treating provider or self	Any	Cancer diagnosis in past 2 y	N/A
Zhou, 2017	Retrospective	197 enrolled; 160 (81%) retained	Clinical	Treating provider	Any stage, NSCLC	Prior to S_x	Ultimately receiving lobectomy, BMI ≥28 kg/m^2, FEV_1% ≤60%, COPD, asthma, or airway hyper reactivity
Zvinovski, 2021	Prospective single-arm	25 enrolled; 18 (72%) retained	Clinical	Treating provider	Early-stage, breast	Within 18 mo of T_x	N/A

Abbreviations: BMI, body mass index; COPD, chronic obstructive pulmonary disease; CT, chemotherapy; CVD, cardiovascular disease; DLCO, diffusion capacity for carbon monoxide; FEV_1, forced expiratory volume in one second; HSCT, hematopoietic stem cell transplantation; KPS, Karnofsky Performance Scale; mo, months; NSCLC, non-small cell lung cancer; RCT, randomized controlled trial; RT, radiotherapy; S_x, surgery; T_x, treatment; VO_2peak, peak volume of oxygen consumption; WHO, World Health Organization; wks, weeks; y, years;

Supplemental Material Table 3: Program structure and components

					Education Content					Other Components					Assessments at Program Entry								Program Staff				
**Publication**	**Pre vs re-habilitation**	**Length (wks)**	**Supervised Aerobic Ex R_x**	**Supervised Resistance Ex R_x**	**Physical activity**	**Nutrition**	**Weight management**	**Psychosocial**	**Cancer specific**	**CVD risk management**	**Tobacco cessation**	**Nutrition intervention**	**Psychosocial counselling**	**Referral to other health professionals**	**Physical activity**	**Dietary practice**	**Tobacco use**	**Physical assessment**	**Cancer-specific**	**Blood pressure**	**Anthropometrics**	**Aerobic fitness**	**Physician**	**Exercise professional**	**Oncologist**	**Registered dietitian**	**Mental health professional**
Baimas-George, 2020	Prehab	12-16	**F:** Daily*<br>**I:** NR<br>**T:** NR<br>**T:** NR	**F:** Daily<br>**I:** NR<br>**T:** NR<br>**T:** NR	✓	✓	?	✓	X	X	?	✓	✓	X	X	✓	X	✓	✓	X	✓	X	✓	?	✓	✓	✓
Berglund, 1994	Rehab	7	**F:** 1x/wk for 4 wks<br>**I:** NR<br>**T:** NR<br>**T:** NR	**F:** 1x/wk for 4 wks<br>**I:** NR<br>**T:** NR<br>**T:** NR	X	✓	?	✓	✓	X	X	X	X	X	✓	X	X	X	✓	X	X	X	?	?	✓	?	?
Bertheussen, 2012	Rehab	8-12	**F:** 10x/wk<br>**I:** 3-4 RPE 2+x/wk + 5-7 RPE 2+x/wk<br>**T:** 60-120 min<br>**T:** Walking, swimming, spinning	None	✓	✓	?	✓	✓	X	X	X	X	X	✓	X	✓	✓	✓	X	✓	✓	✓	✓	✓	✓	✓
Bonsignore 2017, Dolan, 2018	Rehab	22-26	**F:** 1x/wk*<br>**I:** 60-80% VO_2 reserve<br>**T:** NR<br>**T:** Walking, jogging	**F:** 1x/wk*<br>**I:** Varied<br>**T:** 1-3 sets x 10-15 reps<br>**T:** 10-12 whole-body exercises	✓	✓	?	X	✓	X	✓	X	X	X	X	X	✓	?	✓	✓	✓	✓	X	✓	✓	X	X

Supplemental Material Table 3 continued: Program structure and components

Publication	Pre vs re-habilitation	Length (wks)	Supervised Aerobic Ex R_x	Supervised Resistance Ex R_x	Education Content					Other Components					Assessments at Program Entry								Program Staff				
					Physical activity	Nutrition	Weight management	Psychosocial	Cancer specific	CVD risk management	Tobacco cessation	Nutrition intervention	Psychosocial counselling	Referral to other health professionals	Physical activity	Dietary practice	Tobacco use	Physical assessment	Cancer-specific	Blood pressure	Anthropometrics	Aerobic fitness	Physician	Exercise professional	Oncologist	Registered dietitian	Mental health professional
Bonsignore, 2018	Rehab	26	**F:** 1 x/wk*<br>**I:** 60-80% VO_2peak<br>**T:** up to 60 min<br>**T:** Walking, jogging, cycling	**F:** 1x/wk* introduced in wk 8<br>**I:** NR<br>**T:** NR<br>**T:** 10 whole-body exercises	✓	✓	?	✓	X	?	?	X	X	X	✓	X	?	✓	X	✓	✓	✓	?	✓	✓	?	X
Boujibar, 2017	Prehab	Varied	**F:** 3-5x/wk<br>**I:** NR<br>**T:** 45 min<br>**T:** Cycle ergometer	**F:** 3-5x/wk<br>**I:** 70% 1RM<br>**T:** NR<br>**T:** NR	?	?	?	?	?	✓	✓	X	X	X	X	X	✓	?	✓	✓	✓	✓	?	?	?	?	?
Bousquet-Dion, 2017 (Prehab)	Prehab	4	**F:** 3-4x/wk*<br>**I:** 60-70% HR_{peak}<br>**T:** 30 min<br>**T:** Recumbent stepper, treadmill, walking, cycling, jogging	**F:** 3-4x/wk*<br>**I:** NR<br>**T:** NR<br>**T:** 8 exercises	✓	✓	X	✓	X	X	X	✓	X	X	✓	✓	X	X	X	X	✓	✓	X	✓	X	✓	✓
Bousquet-Dion, 2017 (Rehab)	Rehab	8	**F:** 3-4x/wk*<br>**I:** 60-70% HR_{peak}<br>**T:** 30 min<br>**T:** Walking, cycling, jogging	**F:** 3-4x/wk*<br>**I:** NR<br>**T:** NR<br>**T:** 8 exercises	✓	✓	X	✓	X	X	X	✓	X	X	✓	✓	X	X	X	X	✓	✓	X	✓	X	✓	✓

Supplemental Material Table 3 continued: Program structure and components

					Education Content					Other Components					Assessments at Program Entry								Program Staff				
**Publication**	**Pre vs re-habilitation**	**Length (wks)**	**Supervised Aerobic Ex R_x**	**Supervised Resistance Ex R_x**	**Physical activity**	**Nutrition**	**Weight management**	**Psychosocial**	**Cancer specific**	**CVD risk management**	**Tobacco cessation**	**Nutrition intervention**	**Psychosocial counselling**	**Referral to other health professionals**	**Physical activity**	**Dietary practice**	**Tobacco use**	**Physical assessment**	**Cancer-specific**	**Blood pressure**	**Anthropometrics**	**Aerobic fitness**	**Physician**	**Exercise professional**	**Oncologist**	**Registered dietitian**	**Mental health professional**
Bousquet-Dion, 2017 (Pre+Rehab)	Pre + rehab	16	**F:** 3-4x/wk*<br>**I:** Moderate, 60-70% HRmax<br>**T:** 30 min<br>**T:** Recumbent stepper, treadmill, walking, cycling, jogging	**F:** 3-4x/wk*<br>**I:** NR<br>**T:** NR<br>**T:** 8 exercises	✓	✓	X	✓	X	X	X	✓	X	X	✓	✓	X	X	X	X	✓	✓	X	✓	X	✓	✓
Bradley, 2013	Pre + rehab	Varied	**F:** 2x/wk<br>**I:** 60% of max capacity<br>**T:** Portion of 60 min session<br>**T:** NR	**F:** 2x/wk<br>**I:** 60% of max capacity<br>**T:** Portion of 60 min session<br>**T:**	?	✓	?	?	✓	✓	✓	✓	X	X	X	✓	✓	?	X	✓	✓	✓	✓	✓	✓	✓	X
Choi, 2021	Rehab	4-5	**F:** 3x/wk<br>**I:** NR<br>**T:** NR<br>**T:** Walking, biking	**F:** 3x/wk<br>**I:** NR<br>**T:** NR<br>**T:** Whole body	?	✓	X	X	?	X	✓	X	X	X	X	X	✓	?	✓	X	✓	✓	?	?	?	?	?
Dennett, 2019	Rehab	NR	1 hr total but R_x	NR	✓	✓	?	?	X	X	?	X	X	X	X	X	?	?	?	?	?	?	?	✓	✓	✓	?

Supplemental Material Table 3 continued: Program structure and components

					Education Content					Other Components					Assessments at Program Entry								Program Staff				
**Publication**	**Pre vs re-habilitation**	**Length (wks)**	**Supervised Aerobic Ex R_x**	**Supervised Resistance Ex R_x**	**Physical activity**	**Nutrition**	**Weight management**	**Psychosocial**	**Cancer specific**	**CVD risk management**	**Tobacco cessation**	**Nutrition intervention**	**Psychosocial counselling**	**Referral to other health professionals**	**Physical activity**	**Dietary practice**	**Tobacco use**	**Physical assessment**	**Cancer-specific**	**Blood pressure**	**Anthropometrics**	**Aerobic fitness**	**Physician**	**Exercise professional**	**Oncologist**	**Registered dietitian**	**Mental health professional**
Doganay, 2020; Halliday, 2020; Halliday, 2021	Prehab	Varied	**F:** 600-1200 MET-min/wk† **I:** Moderate to vigorous **T:** 150-300 min/wk **T:** NR	Included, but R_x NR†	✓	✓	?	✓	X	X	✓	✓	X	X	✓	✓	X	X	?	X	?	✓	X	✓	✓	✓	?
Gaskin, 1989; Gordon, 2005 (STRETCH)	Rehab	8	None	**F:** NR **I:** NR **T:** 20 min **T:** NR	?	X	X	X	?	X	X	X	X	X	X	X	✓	?	✓	?	✓	X	✓	✓	✓	X	✓
Glattki, 2012	Rehab	4	**F:** 3-5x/wk* **I:** NR **T:** 20-25 min **T:** Cycling	**F:** 2x/wk **I:** 60% of 1-RM **T:** 3 sets x 15 reps **T:** NR	✓	X	X	X	✓	X	✓	X	X	X	X	X	?	✓	✓	?	?	✓	✓	?	?	X	X
Goldsmith, 2020	Prehab	2-4	**F:** 2x/wk* **I:** 60-70% HR_{peak} **T:** 70 mins **T:** Cycle ergometer, walking	None	?	?	X	X	?	?	✓	X	X	X	X	X	✓	✓	✓	?	?	✓	X	✓	?	?	?
Gordon, 2005 (DAART)	Rehab	6	None	Included, but R_x NR†	?	?	?	?	?	?	?	X	X	X	X	X	✓	?	✓	?	✓	?	X	✓	X	X	X

Supplemental Material Table 3 continued: Program structure and components

					Education Content					Other Components					Assessments at Program Entry								Program Staff				
**Publication**	**Pre vs re-habilitation**	**Length (wks)**	**Supervised Aerobic Ex R_x**	**Supervised Resistance Ex R_x**	**Physical activity**	**Nutrition**	**Weight management**	**Psychosocial**	**Cancer specific**	**CVD risk management**	**Tobacco cessation**	**Nutrition intervention**	**Psychosocial counselling**	**Referral to other health professionals**	**Physical activity**	**Dietary practice**	**Tobacco use**	**Physical assessment**	**Cancer-specific**	**Blood pressure**	**Anthropometrics**	**Aerobic fitness**	**Physician**	**Exercise professional**	**Oncologist**	**Registered dietitian**	**Mental health professional**
Hanssens, 2011	Rehab	12	**F:** 3x/wk<br>**I:** NR<br>**T:** NR<br>**T:** Walking, biking, rowing	**F:** 3x/wk<br>**I:** NR<br>**T:** NR<br>**T:** NR	X	✓	X	✓	✓	X	X	X	✓	X	X	X	X	X	✓	X	X	✓	?	✓	✓	X	✓
Heim, 2007	Rehab	Varied	**F:** NR*<br>**I:** NR<br>**T:** NR<br>**T:** Walking	**F:** NR*<br>**I:** NR<br>**T:** NR<br>**T:** 9 exercises	✓	?	?	✓	?	X	X	X	X	X	✓	X	X	X	✓	X	✓	✓	?	✓	X	X	?
Janssen, 2017	Rehab	12	**F:** 2x/wk<br>**I:** 70% peak workload<br>**T:** 20 min<br>**T:** Treadmill, cycle ergometer	**F:** 2x/wk<br>**I:** NR<br>**T:** ≤70 min<br>**T:** NR	✓	X	X	✓	X	?	X	✓	X	✓	X	X	✓	X	✓	X	✓	✓	✓	✓	✓	✓	✓
Kirkham, 2016	Rehab	12	**F:** 2x/wk<br>**I:** 40-80% HRR<br>**T:** 20-30 min<br>**T:** Treadmill, recumbent cycle ergometer, arm ergometer, elliptical, NuStep	**F:** 2x/wk<br>**I:** 11-14 RPE<br>**T:** 15-25 min, 1-3 sets x 8-12 reps<br>**T:** 2-8 exercises	✓	✓	X	✓	✓	?	X	X	X	X	✓	X	✓	X	✓	✓	✓	✓	✓	✓	✓	✓	X

Supplemental Material Table 3 continued: Program structure and components

					Education Content					Other Components					Assessments at Program Entry								Program Staff				
Publication	Pre vs re-habilitation	Length (wks)	Supervised Aerobic Ex R_x	Supervised Resistance Ex R_x	Physical activity	Nutrition	Weight management	Psychosocial	Cancer specific	CVD risk management	Tobacco cessation	Nutrition intervention	Psychosocial counselling	Referral to other health professionals	Physical activity	Dietary practice	Tobacco use	Physical assessment	Cancer-specific	Blood pressure	Anthropometrics	Aerobic fitness	Physician	Exercise professional	Oncologist	Registered dietitian	Mental health professional
Korstjens, 2006; Korstjens, 2007; Velthuis, 2012	Rehab	12	**F:** 2x/wk<br>**I:** NR<br>**T:** 120 min<br>**T:** Cycle ergometer	**F:** 2x/wk<br>**I:** NR<br>**T:** NR<br>**T:** NR	X	✓	X	✓	✓	X	X	X	✓	X	X	X	?	?	✓	X	X	X	?	✓	✓	X	✓
Kröz, 2017	Rehab	10	**F:** 8 sessions over 10 wks*<br>**I:** NR<br>**T:** 45 min<br>**T:** NR	None	✓	X	X	✓	X	✓	?	X	✓	X	X	X	?	?	✓	✓	✓	✓	?	?	?	X	✓
Marhic, 2018	Prehab	NR	**F:** Daily<br>**I:** NR<br>**T:** NR<br>**T:** Cycle ergometer	**F:** Daily<br>**I:** NR<br>**T:** NR<br>**T:** Trunk and upper extremity exercises	X	✓	✓	✓	?	X	✓	X	X	✓	X	X	✓	?	X	X	✓	✓	?	?	?	?	?
Morris, 2009	Rehab	8-12	**F:** 2-3x/wk<br>**I:** 12-14 RPE<br>**T:** Variable<br>**T:** Treadmill, cycle ergometer	**F:** 2-3x/wk<br>**I:** NR<br>**T:** NR<br>**T:** Sliding incline board	✓	✓	X	✓	✓	X	X	X	X	X	X	X	?	✓	X	✓	✓	✓	X	✓	X	X	X

Supplemental Material Table 3 continued: Program structure and components

Publication	Pre vs re-habilitation	Length (wks)	Supervised Aerobic Ex R_x	Supervised Resistance Ex R_x	Education Content: Physical activity	Education Content: Nutrition	Education Content: Weight management	Education Content: Psychosocial	Education Content: Cancer specific	Other Components: CVD risk management	Other Components: Tobacco cessation	Other Components: Nutrition intervention	Other Components: Psychosocial counselling	Other Components: Referral to other health professionals	Assessments at Program Entry: Physical activity	Assessments at Program Entry: Dietary practice	Assessments at Program Entry: Tobacco use	Assessments at Program Entry: Physical assessment	Assessments at Program Entry: Cancer-specific	Assessments at Program Entry: Blood pressure	Assessments at Program Entry: Anthropometrics	Assessments at Program Entry: Aerobic fitness	Program Staff: Physician	Program Staff: Exercise professional	Program Staff: Oncologist	Program Staff: Registered dietitian	Program Staff: Mental health professional
O'Neill, 2018	Rehab	12	**F:** 0-2x/wk*<br>**I:** 30-45% ⇒ 45-60% HRR<br>**T:** 20-35 min<br>**T:** NR	**F:** 0-2x/wk*<br>**I:** 12RM ⇒ 17RM<br>**T:** 2 sets ⇒ 6 sets<br>**T:** NR	?	✓	?	?	?	X	X	✓	X	X	✓	X	✓	X	X	X	✓	✓	✓	✓	X	✓	✓
Rossen, 2020	Rehab	16	**F:** 2x/wk<br>**I:** 15-16 RPE<br>**T:** 25 min<br>**T:** NR	**F:** 2x/wk<br>**I:** 60-80% 1-RM<br>**T:** 3x12 RM ⇒ 3x8RM<br>**T:** NR	?	✓	?	✓	?	X	✓	X	X	✓	X	X	✓	X	✓	X	✓	X	X	✓	✓	?	?
Rothe, 2018	Rehab	8	**F:** 1x/wk*<br>**I:** NR<br>**T:** NR<br>**T:** NR	**F:** 1x/wk<br>**I:** NR<br>**T:** NR<br>**T:** NR	X	✓	✓	✓	X	X	✓	X	X	✓	X	X	✓	✓	X	✓	✓	✓	?	✓	?	?	✓
Sterzi, 2013	Rehab	3	**F:** 5x/wk<br>**I:** 70-80% of max workload<br>**T:** Up to 30 min<br>**T:** Cycle ergometer	**F:** 5x/wk<br>**I:** NR<br>**T:** NR<br>**T:** NR	X	✓	X	✓	✓	X	X	X	X	X	X	X	?	?	X	X	✓	✓	?	?	?	?	?
Wu, 2021	Prehab	Varied	None	**F:** 2x/wk<br>**I:** NR<br>**T:** 8-12 sets<br>**T:** 8 exercises	X	✓	X	✓	X	X	✓	X	X	X	X	✓	✓	?	X	X	X	X	✓	✓	X	X	✓

Supplemental Material Table 3 continued: Program structure and components

					Education Content					Other Components					Assessments at Program Entry								Program Staff				
**Publication**	**Pre vs re-habilitation**	**Length (wks)**	**Supervised Aerobic Ex R_x**	**Supervised Resistance Ex R_x**	**Physical activity**	**Nutrition**	**Weight management**	**Psychosocial**	**Cancer specific**	**CVD risk management**	**Tobacco cessation**	**Nutrition intervention**	**Psychosocial counselling**	**Referral to other health professionals**	**Physical activity**	**Dietary practice**	**Tobacco use**	**Physical assessment**	**Cancer-specific**	**Blood pressure**	**Anthropometrics**	**Aerobic fitness**	**Physician**	**Exercise professional**	**Oncologist**	**Registered dietitian**	**Mental health professional**
Young-McCaughan, 2003	Rehab	12	**F:** 2x/wk*<br>**I:** NR<br>**T:** NR<br>**T:** NR	**F:** 2x/wk*<br>**I:** NR<br>**T:** NR<br>**T:** NR	✓	✓	X	✓	?	?	X	X	X	X	✓	X	X	X	X	X	X	✓	?	✓	?	X	X
Zhou, 2017	Prehab	1	**F:** Daily (7x/wk)<br>**I:** NR<br>**T:** 30 mins<br>**T:** NuSTep	None	✓	X	X	X	X	X	X	X	X	X	X	X	✓	?	X	X	X	X	?	✓	✓	X	X
Zvinovski, 2021	Rehab	14	**F:** 3x/wk*<br>**I:** 60-85% VO_2peak<br>**T:** 45 min by end of program<br>**T:** NR	None	✓	✓	X	X	X	✓	X	X	X	X	✓	X	?	✓	X	✓	✓	✓	?	?	?	?	?

*Indicates a hybrid supervised and unsupervised R_x with unsupervised R_x ranging from 1x/week to daily exercise

† Indicates entirely home-based program

Abbreviations: F, frequency; HRR, heart rate reserve; HR_{peak}, peak heart rate; I, intensity; NR, not reported; RM, repetition maximum; RPE, Borg rating of perceived exertion; T, time or type; VO_2peak, peak volume of oxygen consumption; ⇒, progression; ✓, included; X, not included; ?, cannot determine inclusion;
